# P61 - Atypical presentation of childhood asthma

**DOI:** 10.1186/2045-7022-4-S1-P116

**Published:** 2014-02-28

**Authors:** Selda Ali, Ileana Maria Ghiordanescu, Roxana Silvia Bumbacea

**Affiliations:** 1 Regina Maria Clinic, Constanta, Romania; 2 Elias Emergency Universitary Hospital, Bucharest, Romania; 3 Carol Davila University of Medicine and Pharmacy, Bucharest, Romania

## Background

Food allergy and asthma coexist in many children, although it remains unclear whether or not food allergy and asthma are simply associated to each other due to the underlying predisposition to atopic diseases or whether they are actually causally related.

## Case description

We present the case of a seven years old boy with recurrent episodes of anaphylaxis (4 in one year). Each episode occurred after ingestion of foods containing peanuts and consisted of generalized urticaria and wheezing. 6 months after the last episode he developed persistent asthma symptoms (shortness of breath, cough and wheezing) first time, while running in the park. He was diagnosed with asthma, according to PRACTALL consensus report.

We performed a complete allergological evaluation. The total IgE was elevated. Skin prick tests to main food and aeroallergens were negative, except for peanut where he developed a wheal of 4/5 mm diameter. We measured the specific serum IgE for most common food allergens, peanut was significantly high. Evaluation of lung function revealed that FEV1 was decreased by 27%.

We recommended avoidance measures of all foods containing peanuts. He received treatment with montelukast sodium 5 mg daily and fluticasone propionate 250 mcg daily, and salbutamol as reliever medication and in case of exacerbation or before a physical activity. He did not have any other episode of anaphylaxis, but we could not step down the inhaled corticosteroid. We also prescribed him epinephrine autoinjector.

## Discussion

Children with both food allergy and asthma are at increased risk for severe asthma, particularly if the asthma is uncontrolled. We present the case of an atypical debut of asthma in a child with IgE mediated food allergy. During follow up the evolution of asthma was independent of avoidance measures of the culprit food. The patient might be a candidate for anti-IgE treatment.

